# AmyloGram reveals amyloidogenic potential in stroke thrombus proteomes

**DOI:** 10.1042/BCJ20253317

**Published:** 2025-11-17

**Authors:** Douglas B. Kell, Karen M. Doyle, J. Enrique Salcedo-Sora, Alakendu Sekhar, Melanie Walker, Etheresia Pretorius

**Affiliations:** 1 Department of Biochemistry, Cell and Systems Biology, Institute of Systems, Molecular and Integrative Biology, University of Liverpool, Liverpool, L69 7ZB, U.K; 2 The Novo Nordisk Foundation Centre for Biosustainability, Technical University of Denmark, Kongens Lyngby, 2800, Denmark; 3 Department of Physiological Sciences, Faculty of Science, Stellenbosch University, Stellenbosch, 7602, South Africa; 4 Department of Physiology and Galway Neuroscience Centre, School of Pharmacy & Medical Sciences, University of Galway, Ireland; 5 CÚRAM Research Ireland Centre for Medical Devices, University of Galway, Galway, Ireland; 6 Department of Neurology, The Walton Centre, Liverpool, L9 7LJ, U.K; 7 Department of Neurological Surgery, University of Washington, Seattle, WA 98104, U.S.A.

**Keywords:** amyloid, amyloidogenic sequences, atherothrombotic, cardioembolic, fibrinaloid, inflammation, proteomics, stroke

## Abstract

Amyloidogenic proteins play a central role in a range of pathological conditions, yet their presence in thrombi has only recently been recognized. Whether computational prediction tools can identify amyloid- forming potential in thrombus proteomes remains unclear. AmyloGram is a computational tool that estimates amyloid-forming potential based on n-gram sequence encoding and random forest classification. Using AmyloGram, we analyzed 204 proteins in UniProt that were tagged by humans as amyloidogenic. We then applied the same approach to proteins identified in thrombi retrieved using mechanical thrombectomy from patients with cardioembolic and atherothrombotic stroke. In addition, we used AmyloGram to analyze the amyloidogenicity of 83,567 canonical human protein sequences. Among the UniProt-annotated ‘amyloid’ set, nearly all proteins received AmyloGram scores above 0.7, including 23 of the 24 human proteins. Even the lowest-scoring human protein, lysozyme (scoring 0.675), is known to form amyloid under certain conditions. In thrombi from both stroke subtypes in four different studies, all detected proteins (with a single exception) had AmyloGram scores above 0.7, suggesting a high likelihood of amyloid content. A majority of unannotated proteins also achieve AmyloGram scores exceeding 0.7. AmyloGram reliably identifies known amyloid-forming proteins and reveals that stroke thrombi are enriched for proteins with high amyloidogenic potential. These findings support the hypothesis that thrombus formation in stroke involves amyloid-related mechanisms and warrant further investigation using histological and functional validation.

## Introduction

**Figure 1:**
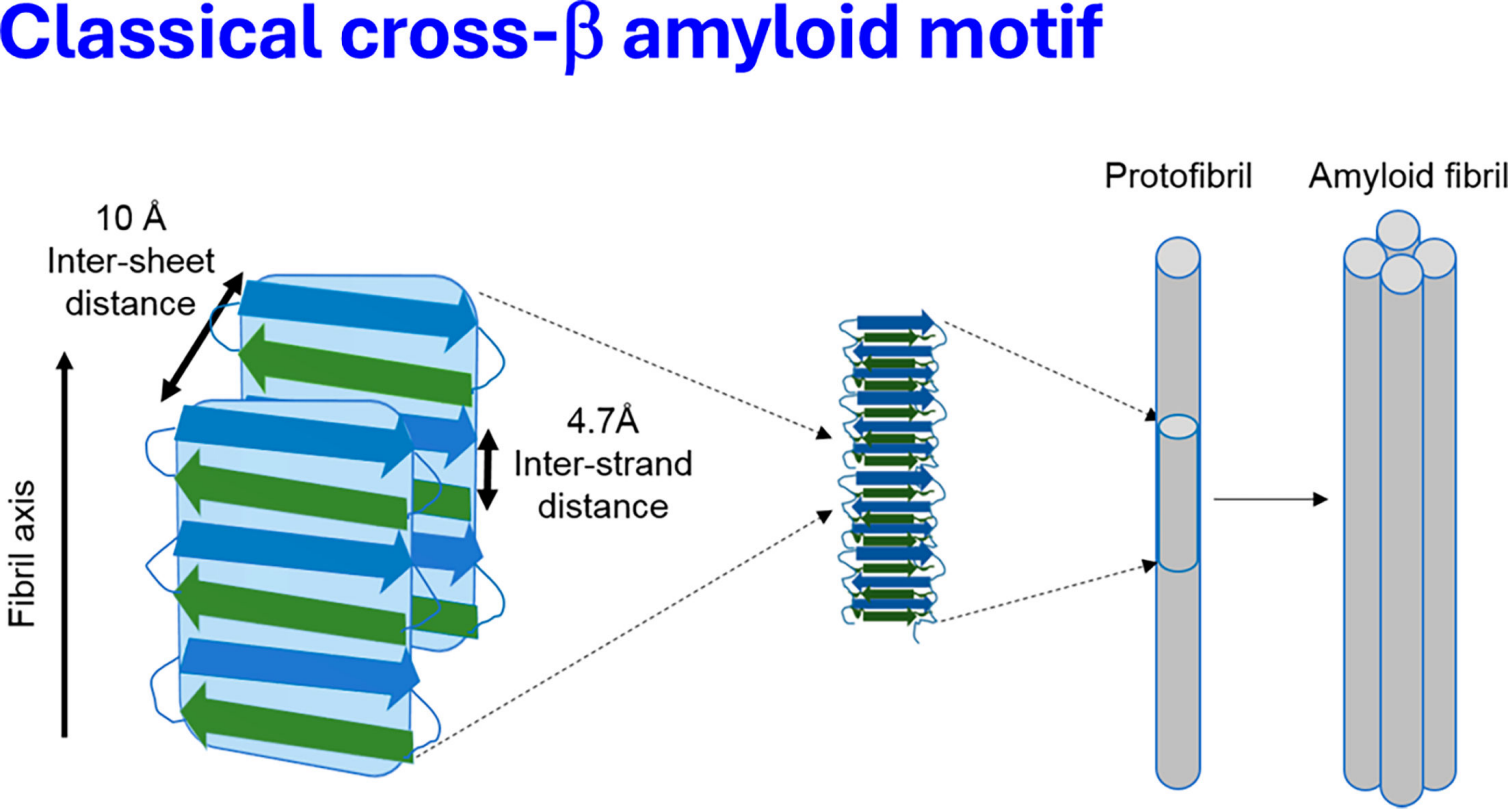
The basic cross-β-sheet structure underlying amyloid proteins. In each sheet, the β-strands (light and dark blue arrows) are aligned perpendicular to the fibrillar axis with a spacing of 4.7 Å and stabilized via hydrogen bonds. (Here these are antiparallel, but in other variants they are parallel.) The cross-β-sheet structure is formed by association of β-sheets at a distance of approximately 10 Å (it can vary from 6 to 12 Å depending on the sidechains involved [37,42]). The further assembly of such cross-β-sheet structures results in the formation of protofibrils, and continued aggregation leads to the mature amyloid fibril. Adapted from a CC-BY 4.0 publication [43] using Biorender.com.

Proteins can adopt multiple stable conformations or macrostates. One important family of such conformations is represented by amyloids, which differ markedly from the native structures synthesized on the ribosome [1–12]. Amyloidogenic proteins, long studied in the context of neurodegeneration and systemic amyloidoses [3,13–18], are increasingly recognized for their roles in vascular and thrombotic pathologies [19–25]. Recent evidence suggests that amyloid proteins may also contribute to clot formation and persistence in ischemic stroke [26,27]. Although ‘irreversibly’ formed amyloids (see [28]) are typically separated from native forms by a high energy barrier [29–34], they are often significantly more stable [30,32,35,36] and tend to form insoluble fibrils that can oligomerize. These properties are conferred by a defining structural feature, the cross-β motif [8,37–41], which consists of β-sheets aligned perpendicular to the fibril axis. These sheets typically form stacks stabilized by hydrogen bonds and can assemble into protofibrils and eventually mature amyloid fibrils (Figure 1).

Almost any protein is capable of forming amyloid structures [42,44], provided it contains a short run of amyloidogenic sequences [45,46]. The SARS-CoV-2 spike protein provides a highly pertinent example [47–49], with its demonstrated ability both to form amyloid and to cross-seed amyloid formation [50], including during blood clotting [51,52].

Given the diversity of sequences capable of forming amyloid, numerous computational tools (31 were recently summarized in [24]) have been developed to predict amyloidogenic potential based on sequence features. In this study, we focus on AmyloGram [53,54], a predictive algorithm that uses n-gram encoding of amino acid sequences and a random forest [55] classifier to assign a single score between 0 and 1, reflecting overall amyloid-forming potential. In addition to a global score, the server provides residue-level predictions across the protein sequence, allowing for regional analysis of amyloidogenicity. Although comparisons with multiple prediction methods would be a worthwhile separate undertaking, the present study focused exclusively on AmyloGram for three reasons: (i) it demonstrated the highest predictive performance in its original validation [53]; (ii) it provides a single, interpretable score ranging from 0 to 1, which is independently verified in the present analysis; and (iii) it is available (see materials and methods) both as a web application and as R code. The principal sequence determinants used by AmyloGram to predict amyloidogenicity are hydrophobicity, β-sheet propensity, and reduced amino acid flexibility [53].

The present study aimed to assess computationally whether proteins identified in ischemic stroke thrombi exhibit high levels of amyloid-forming potential consistent with the presence of amyloid as observed histologically, as previous work has demonstrated amyloid content in thrombi retrieved by mechanical thrombectomy from patients with acute ischemic stroke (AIS) [26,27]. Based on these findings, we hypothesized that stroke thrombi are enriched in proteins with high intrinsic amyloidogenicity. To test this, we used AmyloGram as a validated computational prediction tool to assess amyloid potential across known amyloidogenic proteins and thrombus proteomes. A sequence-based predictive approach was selected rather than a structure-based method such as Aggrescan 3D [56,57]. Sequence information is generally sufficient (i) to direct protein folding [58] and (ii) to enable accurate three-dimensional structure prediction [59–63]. In addition, Aggrescan 3D requires a PDB entry, and many of the proteins analyzed in this study lack corresponding structural data.

Overall, the findings provide insight into possible molecular contributors to amyloid-driven clot formation and stability in the context of cerebrovascular disease. A preprint has been lodged [64].

## Results

### AmyloGram scores across verified amyloid proteins

**Figure 2:**
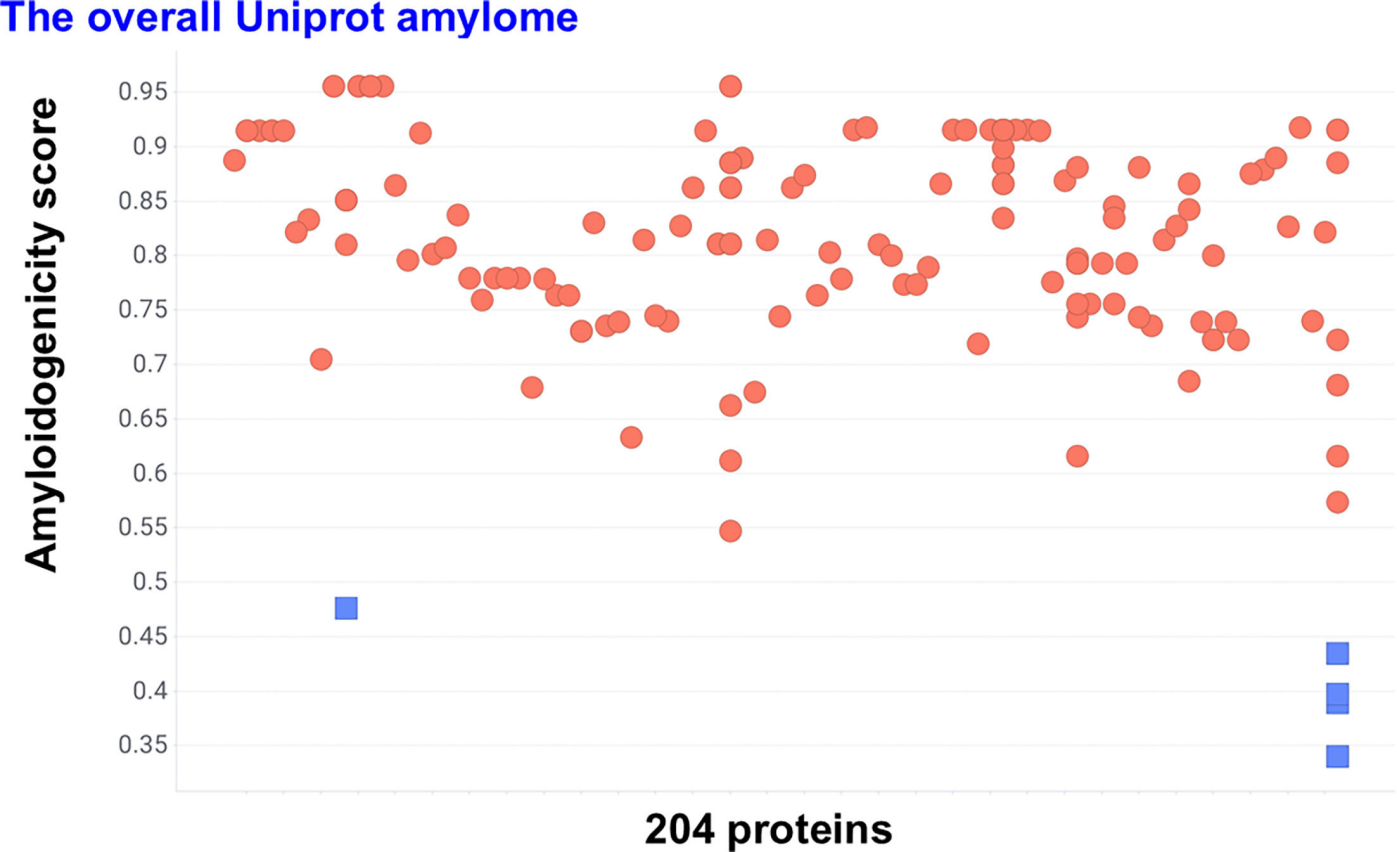
AmyloGram-predicted amyloidogenicity scores for 204 proteins annotated as amyloidogenic in UniProt. Each protein was assigned a global score between 0 and 1 based on sequence features. Proteins scoring below 0.5 are indicated by blue squares. The majority of proteins scored above 0.7, consistent with experimentally verified amyloid-forming potential.

A total of 204 proteins were retrieved from UniProt using the ‘reviewed’ plus ‘amyloid’ keyword filters. These represented 95 species and included 24 human proteins. The most frequent entry was the major prion protein, which accounted for 70 of the 204 sequences. All 204 sequences were submitted to AmyloGram, and global amyloidogenicity scores were obtained. Five proteins received AmyloGram scores below 0.5. The remaining proteins showed a range of scores, with the great majority scoring above 0.7 (Figure 2). Note that the median score was 0.8716.

### AmyloGram scores in human amyloid proteins

**Figure 3:**
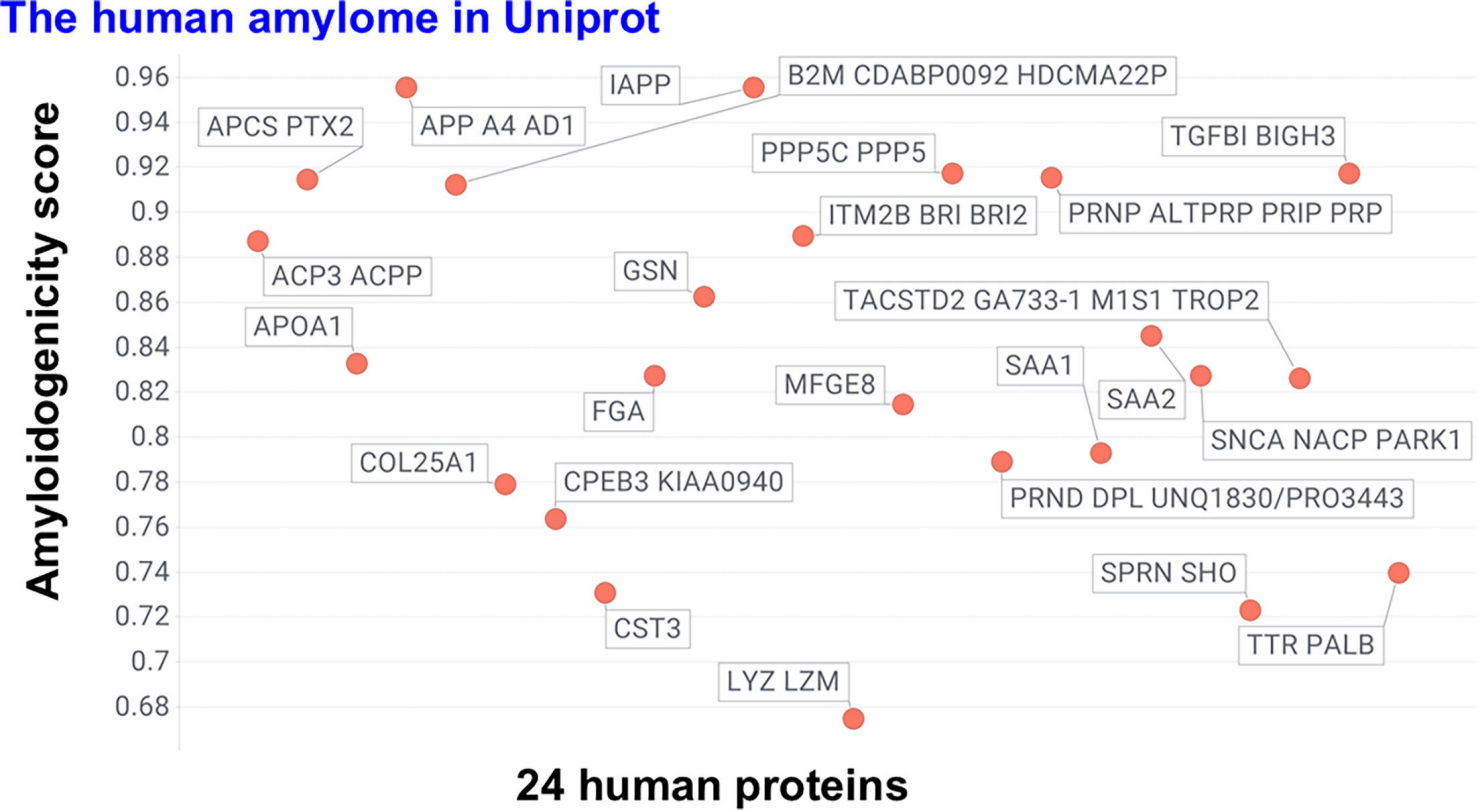
AmyloGram-predicted amyloidogenicity scores for 24 human proteins annotated as amyloidogenic in UniProt. Global scores are shown for each protein based on full-length canonical sequences. All proteins scored above 0.65, with several exceeding 0.9. Lysozyme, the lowest-scoring protein, still exceeded 0.67 and is known to form amyloid under specific conditions. Seven proteins scored above 0.9. Full names are given in Supplementary Table S1.

Among the 24 human proteins in the dataset, AmyloGram scores ranged from 0.675 to above 0.95 (Figure 3). The highest-scoring proteins included amyloid precursor protein (APP, part of whose sequence forms the highly amyloidogenic Aβ protein [65]) and islet amyloid polypeptide (IAPP or amylin [66–70]), both exceeding 0.95. Other high-scoring proteins included serum amyloid P [71–73], β2-microglobulin [31,74–77], protein phosphatase 5, the major prion protein [78–80], and transforming growth factor β1 [81]. Given its known tendency to form amyloid blood clots [32,82,83] that are rather resistant to fibrinolysis [84,85], fibrinogen A, with a score of 0.8276, is also of notable interest (and see below). Two serum amyloid A isoforms scored 0.7932 and 0.8541.

The four lowest-scoring human proteins that were tagged as amyloid were cystatin C, lysozyme, protein shadoo [86,87], and transthyretin, with lysozyme scoring 0.675 and transthyretin scoring 0.74 (Figure 3). Consequently, we can safely conclude that any protein with a score above 0.7 is likely to be amyloidogenic, whether alone or by cross-seeding. The median score of these 24 was 0.83.

### Amylogram scores in the overall unannotated human proteome

**Figure 4:**
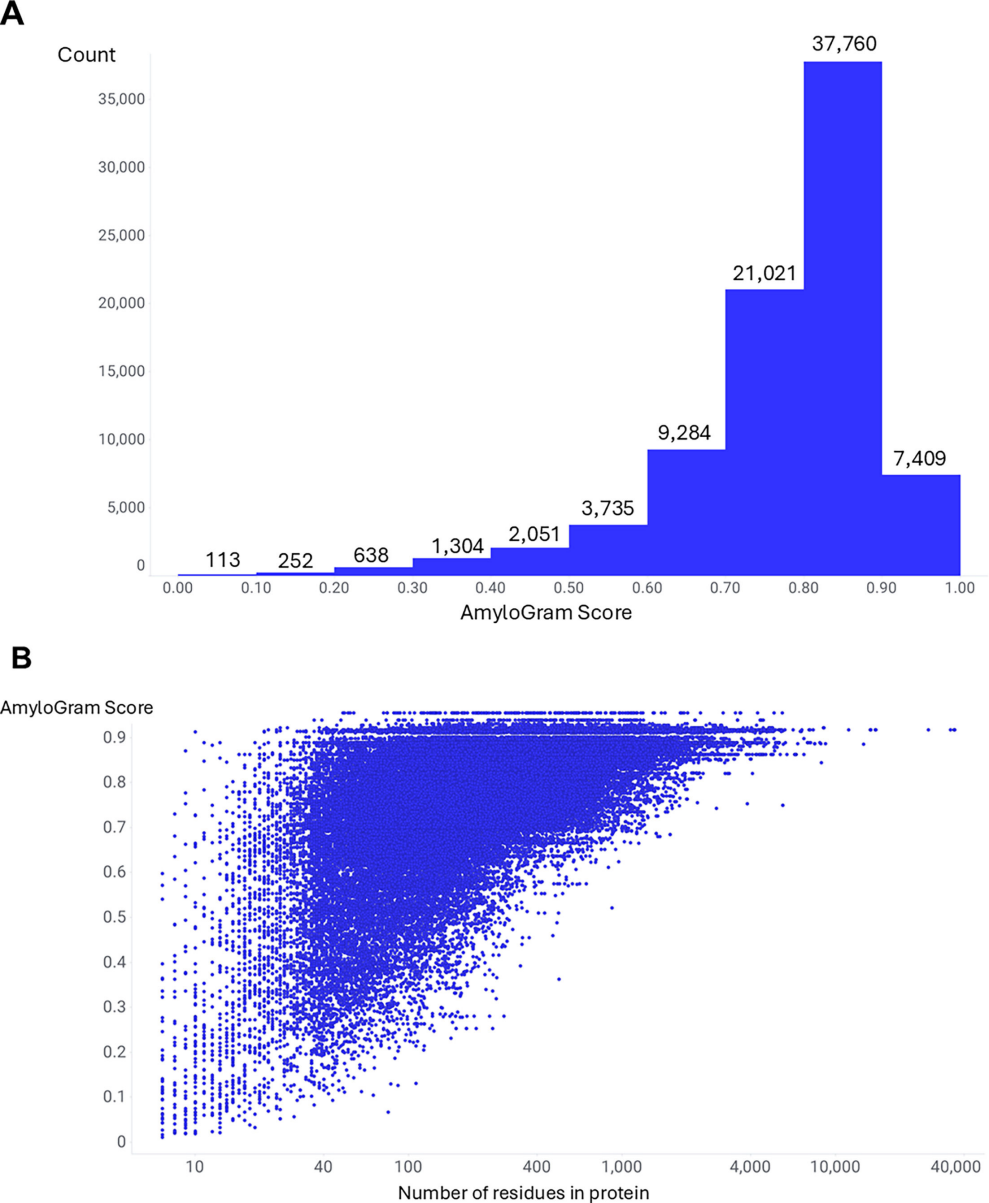
Distribution of AmyloGram scores across 83,567 full-length canonical human polypeptides in UniProt. (**A**) Histogram of score frequency shows that 79.2% of sequences scored above 0.7, suggesting widespread presence of amyloidogenic motifs. (**B**) Scatter plot of score versus polypeptide length in amino acid residues shows a weak positive correlation, possibly reflecting increased opportunity for amyloid-prone motifs in longer proteins. Protein accession numbers and scores are listed in Supplementary Table S2.

Figure 4 shows the distribution of the 83,567 human proteins at UniProt for which Amylogram returned a score (Supplementary Table S2); 66,190 (79.2%) show a value exceeding 0.7 (**Figure 4A**), consistent with the view that ‘most’ proteins can indeed form amyloid under some circumstances. This observation is consistent with the hypothesis that a large fraction of human proteins contain local amyloidogenic segments and that sequence-based prediction tools such as AmyloGram are capturing real biochemical potential rather than overpredicting. There is also a minor tendency (Figure 4B, r^2^ ~ 0.36 in lin–log co-ordinates, just 0.09 in lin–lin, neither line displayed) towards an increase in amyloidogenicity with length.

### AmyloGram scores in stroke thrombi by subtype

**Figure 5:**
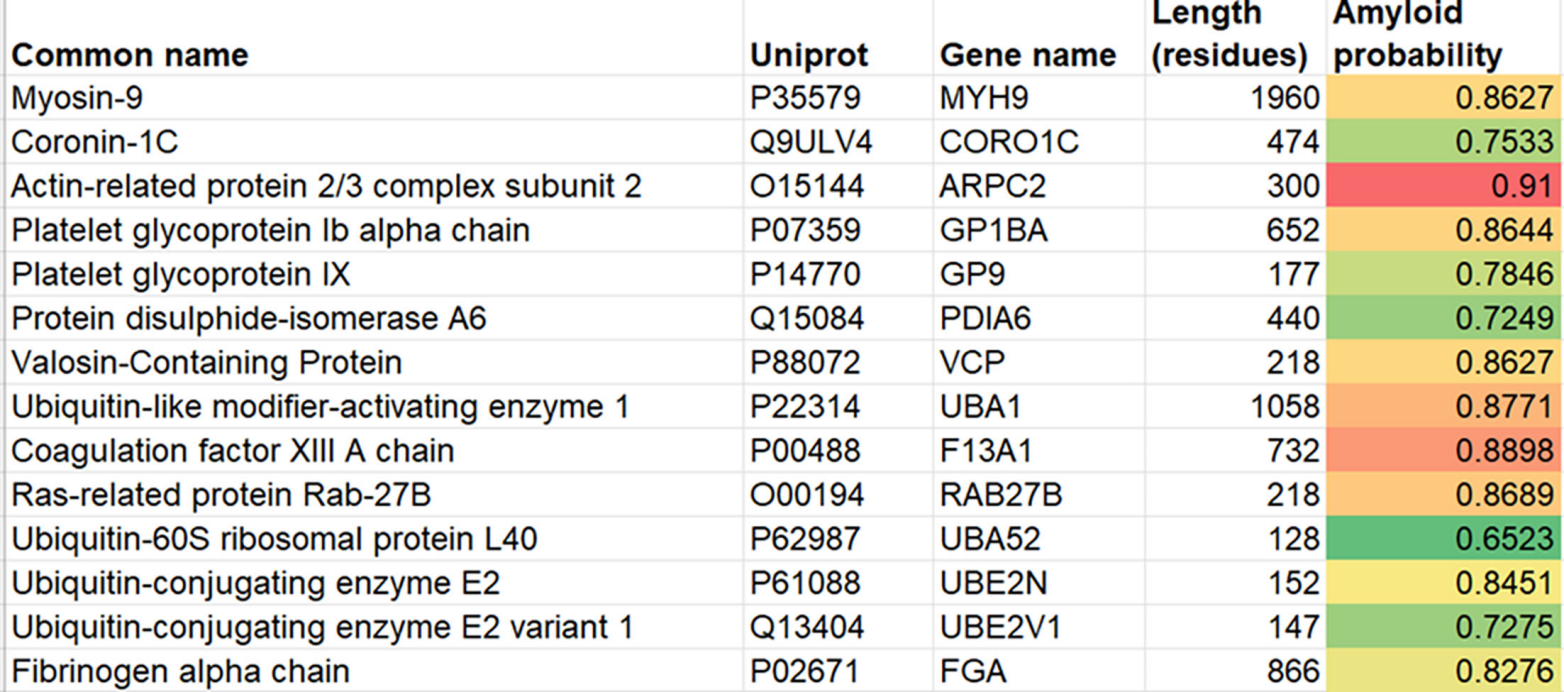
AmyloGram-predicted amyloidogenicity scores for 14 proteins identified in thrombi from patients with cardioembolic stroke, as reported in Table 1 of Rossi et al. [88]. Each protein was scored using its full-length sequence, and all received global scores above 0.7. Color shading reflects the magnitude of the predicted amyloidogenicity.

We recently established that the thrombi removed by mechanical thrombectomy from patients who have suffered an ischemic stroke also contain abundant amounts of amyloid material [26,27]. In a previous strategy [25], we asked the question as to whether proteins additional to fibrinogen components that are known to occur in amyloid clots [24] can be used to predict the likely amyloid nature of those clots even when their amyloid status had not previously been measured. We here extend this strategy by asking the related question as to whether proteins observed experimentally (using proteomics) in stroke thrombi are also amyloidogenic as judged by AmyloGram. We first analyzed proteins identified in thrombi from two published proteomic studies of ischemic stroke. The first study reported 14 proteins enriched in thrombi from patients with cardioembolic stroke [88]. All 14 proteins received AmyloGram scores greater than 0.7 (Figure 5).

**Figure 6:**
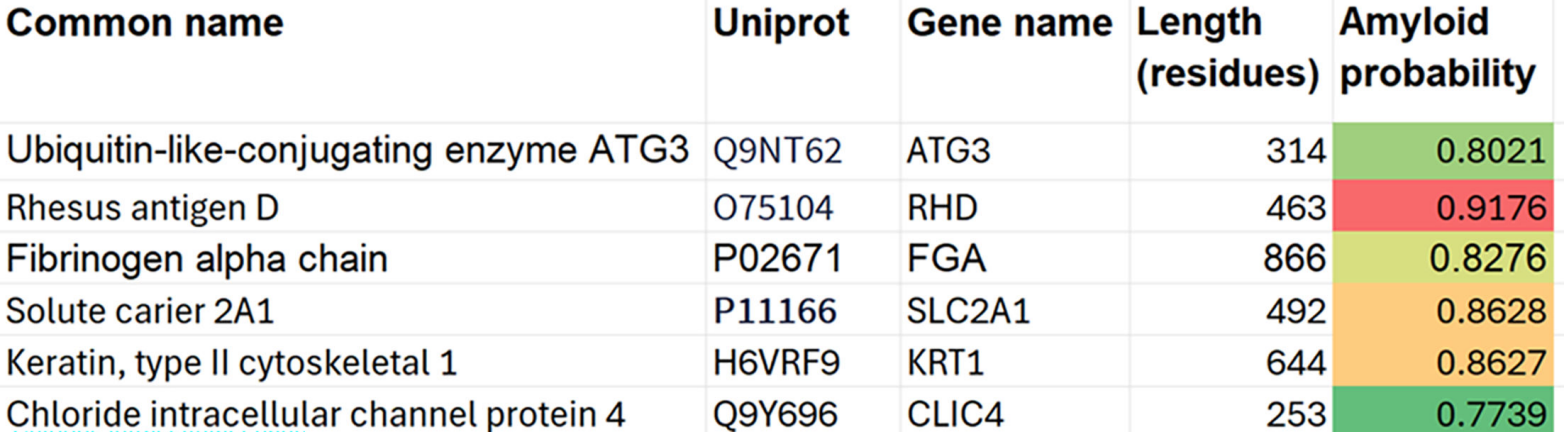
AmyloGram-predicted amyloidogenicity scores for six proteins reported to be more abundant in thrombi from patients with atherothrombotic stroke, based on the dataset from Lopez-Pedrera et al. [89]. All proteins received global scores above 0.75. Color shading relates to the predicted amyloidogenicity score.

The second study compared thrombi from cardioembolic and atherothrombotic stroke and identified six proteins with higher abundance in the atherothrombotic group [89]. These proteins were ATG3, RHD, FGA, SLC2A1, KRT1, and CLIC4. All six received AmyloGram scores greater than 0.75 (Figure 6).

**Figure 7:**
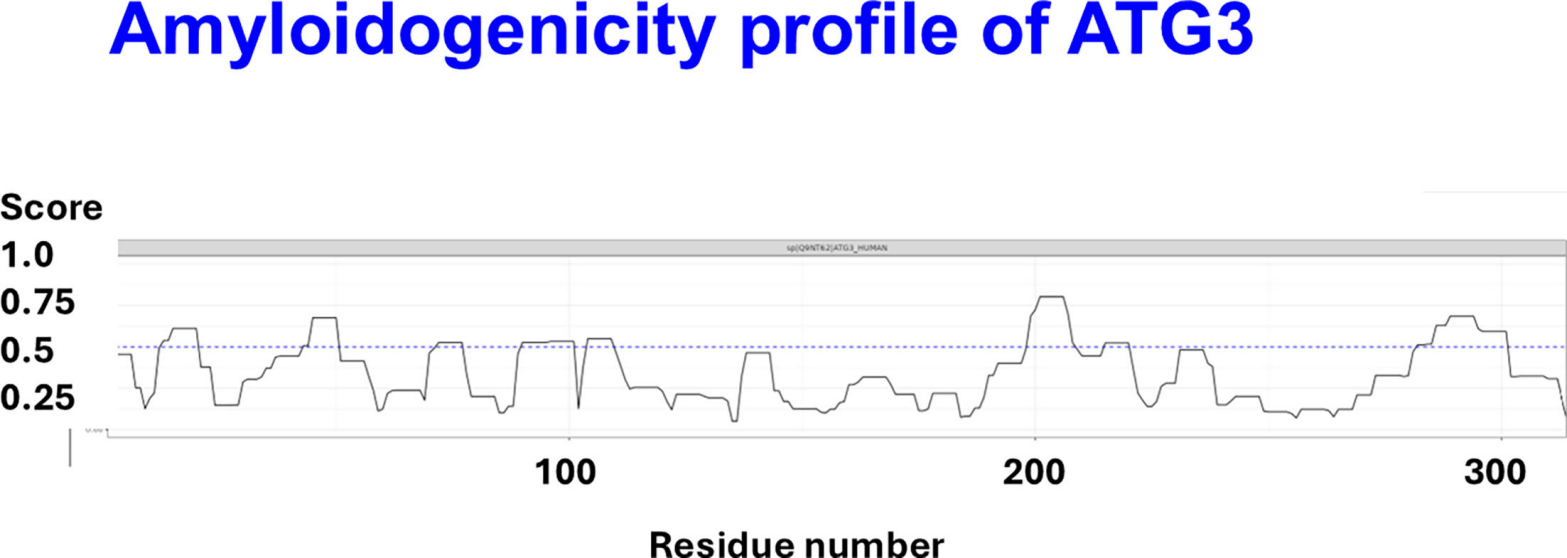
Residue-level amyloidogenicity profile of ATG3, generated using the AmyloGram server (accessed June 28, 2025). The plot displays predicted amyloidogenicity across the amino acid sequence, with several regions exceeding 0.5 and one run exceeding 0.75. This highlights localized segments with high fibril-forming potential within the full-length protein.

Although far from the most amyloidogenic of these proteins, a residue-level amyloidogenicity profile for ATG3 is shown in Figure 7.

**Figure 8:**
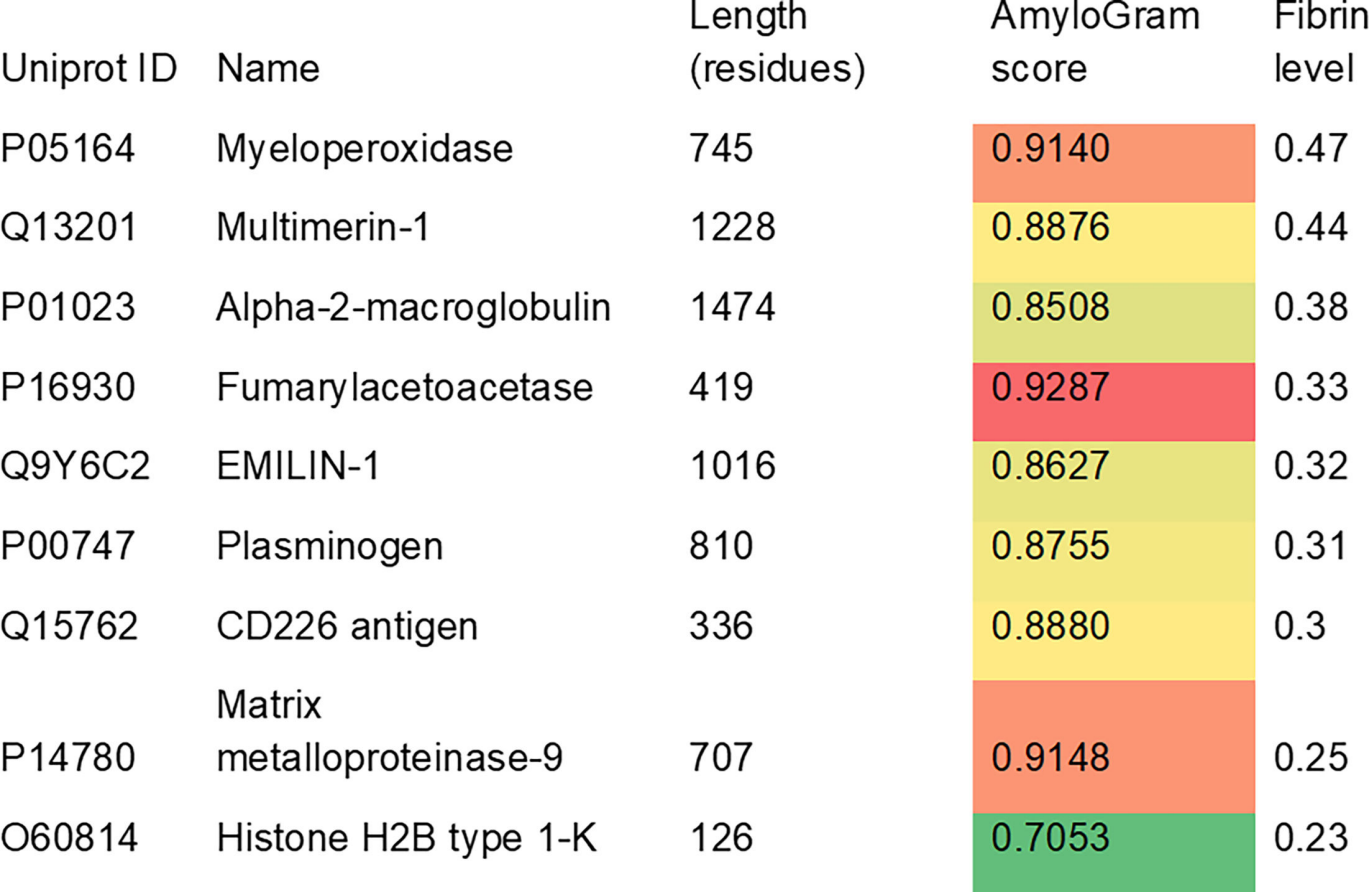
Proteins enriched in ‘fibrin-rich’ clots mechanically thrombectomized from individuals who had suffered an ischemic stroke. Protein names are taken from Figure 1 of [90]. Color shading relates to the predicted amyloidogenicity score.

We also used AmyloGram to analyze the nine proteins listed as being enriched in high-fibrin clots thrombectomized from (mainly cardioembolic) ischemic strokes in a paper by Doche and colleagues ([90]; their Figure 1). The results are given in Figure 8.

Some of these proteins are quite recondite, in that there is no obvious enzymatic reason why a protein such as fumarylacetoacetase (fumarylacetoacetate hydrolase) should be enriched in stroke thrombi (its deficiency can be responsible for hereditary tyrosinemia type I [91–93]). Again, however, every single protein listed in Figure 8 has an AmyloGram score of more than 0.7, with eight of the nine having scores exceeding 0.85. Fumarylaetoacetase is actually the most amyloidogenic of them all, with an AmyloGram score exceeding 0.92. This further reinforces the view that the proteins contained in such clots tend to be highly amyloidogenic in nature, whatever their biochemical or enzymatic functions, and likely because they are cross-seeding each other’s amyloidogenesis [24,25,94].

**Figure 9:**
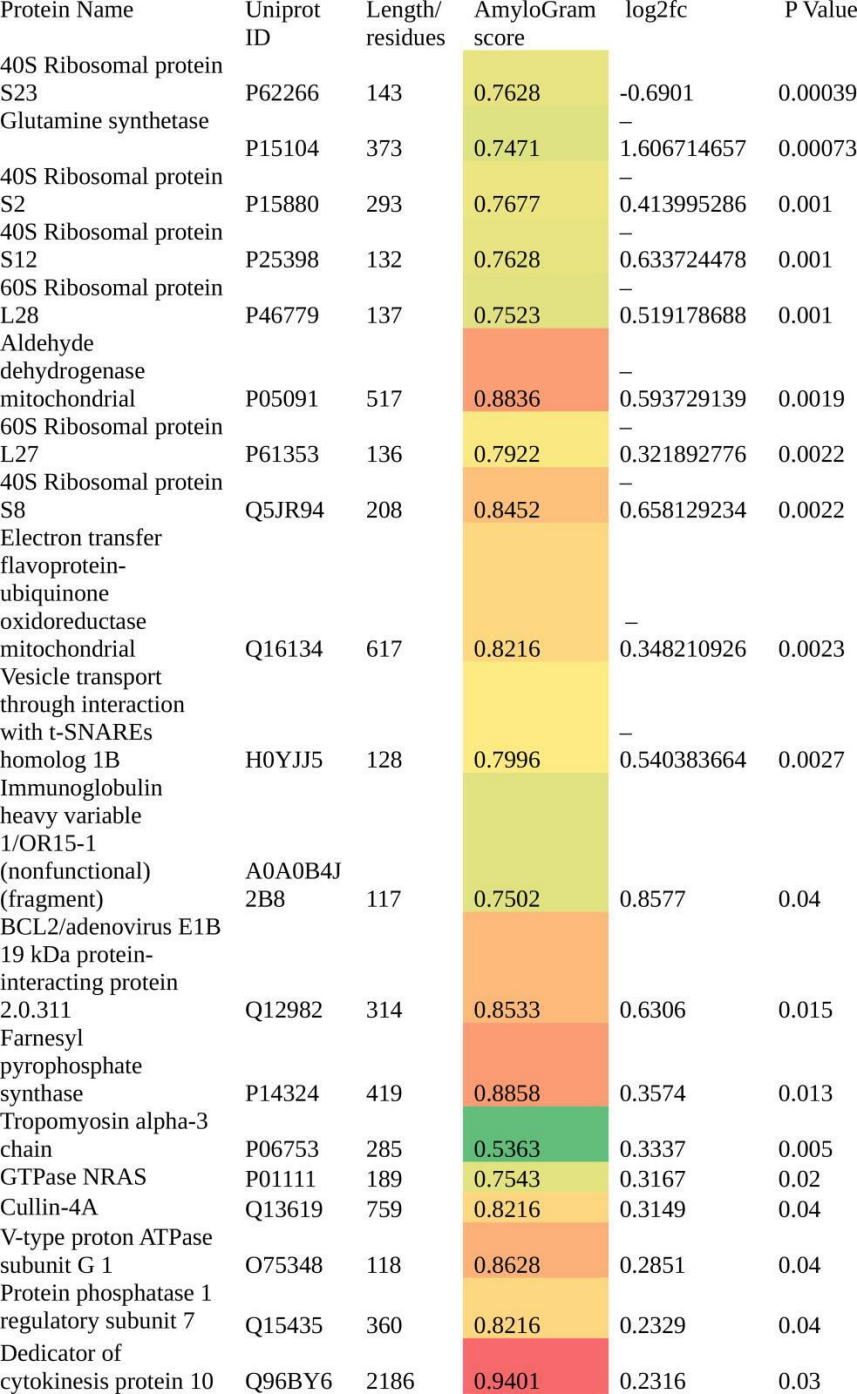
AmyloGram scores of proteins highlighted as being present in thrombi in a study [95] of thrombus perviousness. AmyloGram analyses were performed as in Figures 5, 7, and 8.

We next analyzed the data for 19 proteins tabulated in a paper by Schartz and colleagues [95], who were interested in the proteome content of variably porous thrombi. 17 of the 19 proteins had AmyloGram scores exceeding 0.75 (18/19 exceeded 0.7), and data are displayed in Figure 9.

**Figure 10:**
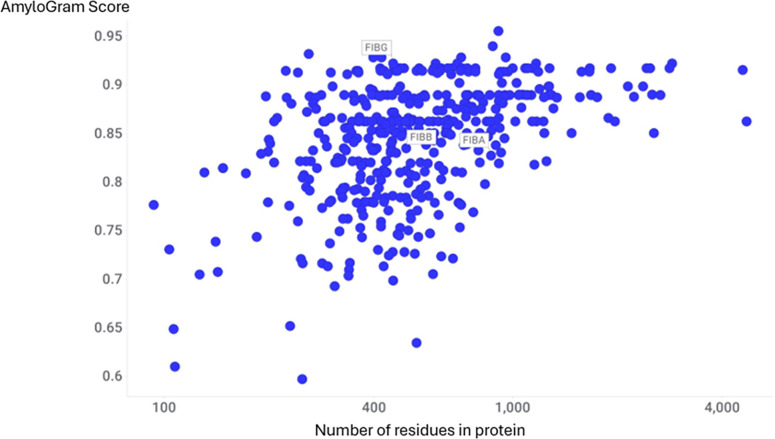
AmyloGram scores and protein lengths for 438 proteins identified by Dargazanli et al*.* [96] as present in thrombi from 32 cardioembolic and 28 atherothrombotic stroke cases. Fibrinogen chains A, B, and G are indicated.

Data from Dargazanli et al. [96], who analyzed the thrombus proteome in 32 cardioembolic and 28 atherothrombotic stroke cases, identified 2455 proteins in total, of which 438 were detected in all samples. Among these, 431 proteins had AmyloGram scores of ≥0.7, and the median value (the 219th protein) lay within a group of 40 proteins (including fibrinogen B) with a score of 0.86. These results, illustrated in Figure 10, demonstrate that the vast majority of proteins consistently present across all 60 thrombi exhibit high predicted amyloidogenic potential.

**Figure 11:**
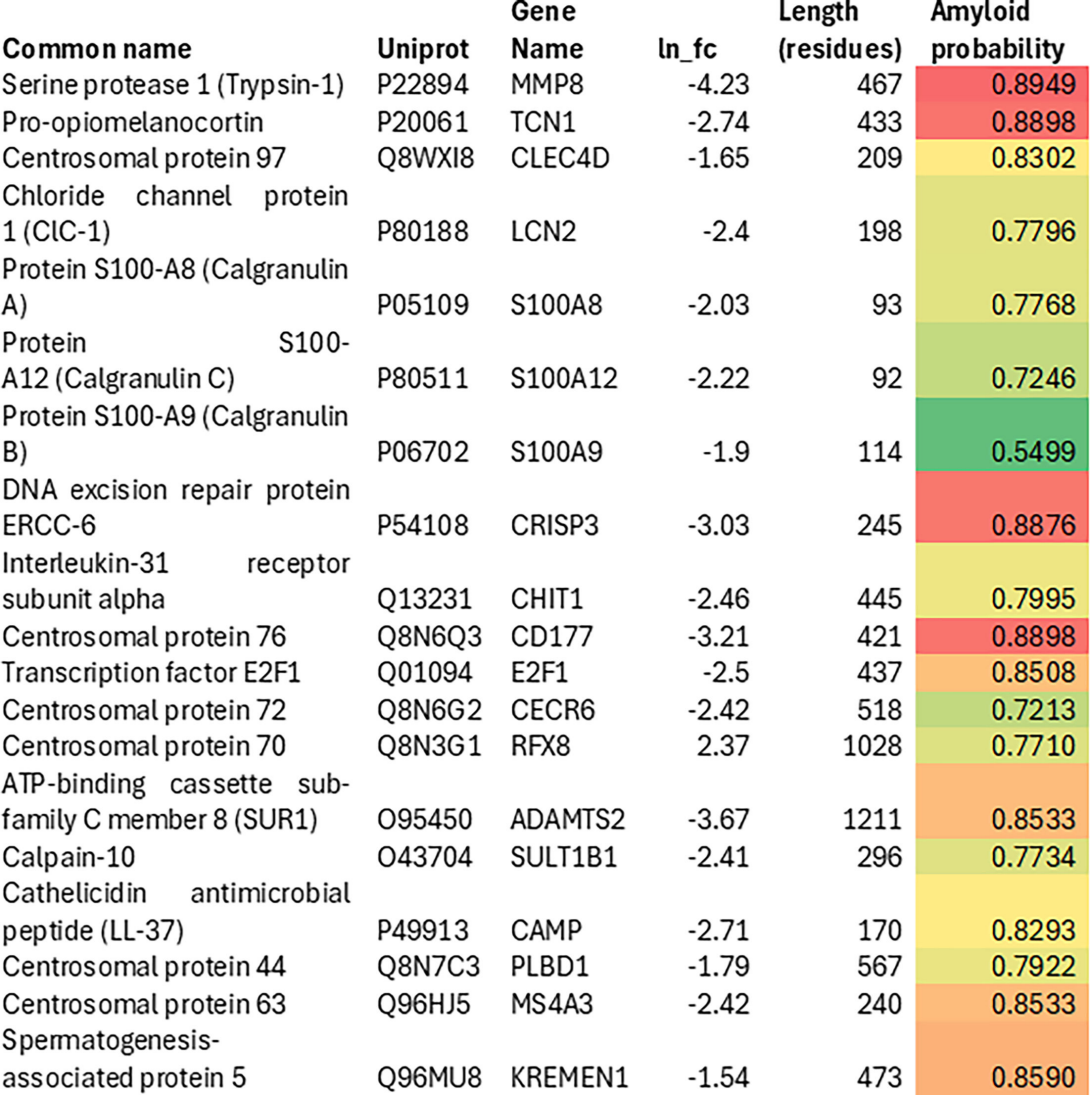
Proteins expressed in thrombi associated with early neurological improvement (negative fold change observed in 18 of the 19 proteins, indicating poorer outcomes) and their corresponding AmyloGram scores. Original data and log-transformed fold-change values (ln fc) were obtained from Figure 4A of [97].

Santo et al. [97] (their Table 4A) examined the relationship between gene expression in thrombi and early neurological improvement, where a decrease in expression denotes a poorer outcome. Figure 11 presents data for 19 proteins, of which all but one had AmyloGram scores exceeding 0.7. The median value, corresponding to the cathelicidin antimicrobial peptide (LL-37) [98], was 0.8293. This dataset provides additional evidence of marked enrichment for amyloidogenic proteins within thrombi, consistent with their association with adverse clinical outcomes.

## Discussion

Thrombi responsible for large vessel occlusion (LVO) in the setting of AIS are commonly characterized by a low recanalization rate, even after intravenous thrombolysis (e.g. [99–108]). Regardless of their cause or variability, brain clots causing LVO have an outer shell and core–shell structure that affects how easily they break down [109–111]. Studies using immunohistology have shown that these clots are quite diverse, with the thrombus outer shell being more resistant to tPA and thrombolysis generally than is the thrombus core [110]. Laboratory studies have also shown that clot shrinkage and compression can increase the clot’s resistance to breakdown [112–116], including by enhancing strong crosslinks between fibrin and other molecules. Unlike the fibrous fibrin in the inner core, the fibrin in the shell can appear dense and matted, and our previous work also demonstrated a tendency towards amyloidogenesis occurring in the shell of the clots examined [26,27].

This study extends that work [26,27] by evaluating whether computational prediction of amyloidogenicity aligns with experimentally verified protein behavior and whether thrombi retrieved from patients with ischemic stroke contain proteins predicted to be amyloid-forming. Using AmyloGram, we analyzed both a reference set of 204 human-reviewed amyloidogenic proteins from UniProt and protein lists derived from thrombus proteomic studies of cardioembolic and atherothrombotic stroke. In all cases, the majority of computationally assessed proteins, and each of those observed experimentally, scored above 0.7 at AmyloGram, consistent with their experimentally confirmed amyloid-forming capacity. The consistency of high amyloidogenicity scores across both stroke subtypes determined here implies that amyloid-related mechanisms may be a shared feature, which drives the crosslinking between fibrin and other molecules, leading to a dense outer shell filled with tightly packed fibrin and platelets that are resistant to thrombolysis.

Among the 24 human proteins in the UniProt-verified dataset, scores ranged from 0.675 to greater than 0.95. Even lysozyme, with the lowest score of the 24 at 0.675, is well known to be capable of amyloidogenesis. Indeed, mutant forms of lysozyme are naturally amyloidogenic [117–119], and lysozymes are widely studied as an amyloidogenic model [118–126], albeit some require a rather acidic pH to create stable amyloid conformations [127]. Cystatin C [128–131], with an AmyloGram score of just 0.73, and transthyretin [132–137] (0.74) are directly responsible for two well-known amyloidoses. These observations strongly support the use of a 0.7 threshold as a conservative predictor of amyloid-forming potential using AmyloGram.

We might have sought a ‘negative’ set of proteins that are not considered to form amyloid experimentally, but in fact, this is not easily done. This is because it is considered that a majority of proteins can potentially form amyloid under suitable conditions [42,44,138,139]; insulin is another well-known human example [140–146], and the existence of a great many well-established amyloidogenic proteins above our nominal threshold of 0.7 is seen as sufficient justification for the approach. Note that AmyloGram does predict many proteins to have much lower amyloidogenicity (Figure 4), so we do not consider that it is pathologically over-predicting. Interestingly, even bacterial species produce proteins with strong amyloidogenic potential [147–151], where these structures may serve physiological roles such as defense or biofilm reinforcement [152].

The thrombus proteomic data demonstrated that every protein identified in three separate studies involving both cardioembolic and atherothrombotic stroke clots received an AmyloGram score above 0.7. While proteomic profiling does not directly confirm the presence of amyloid structures (although it is consistent with a noticeable resistance to the kinds of protease commonly used in proteomics [84,85]), this finding suggests that the protein composition of these thrombi is strongly biased toward sequences with intrinsic amyloidogenic features, regardless of their biochemical activities. These results align with prior histological evidence of amyloid deposition in stroke thrombi, as detected by thioflavin T [26,27], and provide a straightforward explanation for the comparative resistance [153,154] of these clots towards fibrinolytics such as tissue plasmin(ogen activator).

The consistency of high amyloidogenicity scores across both stroke subtypes implies that amyloid-related mechanisms may be a shared feature of thrombus formation or stabilization. The complex proteomes observed in these (and other [23,84,85]) clots are only realistically accounted for by a rather widespread cross-seeding (many papers are summarized in [24,25]). However, subtle differences likely exist, since the observed amyloid dispositions were highly heterogeneous both within and between clots [26,27]. For example, fibrinogen A and serum amyloid A are known to co-aggregate [155] and were among the high-scoring proteins observed in the thrombus datasets. Future work could assess whether amyloid burden or composition varies by stroke mechanism, infarct size, or thrombus resistance to lysis.

The physiological or pathological inducers of aggregation within thrombi likely involve multiple mechanisms. In addition to the extensive cross-seeding discussed above, it is plausible that amyloidogenesis occurs before clot incorporation, with preformed aggregates promoting subsequent accretion. Finally, this work raises the possibility that cross-seeding interactions between fibrin-based clot components and classical amyloid-forming proteins, such as APP, Aβ, or IAPP, may contribute to the persistence or remodeling of thrombi. Such interactions have been observed in other settings [23,156–161] and may link clot composition to post-stroke inflammation or neurodegeneration.

The residue-level profiles from AmyloGram, as illustrated for ATG3 (Figure 6), also suggest that specific regions within proteins (e.g. [34]) may disproportionately contribute to amyloidogenesis. These localized high-scoring segments (‘hotspots’) could represent therapeutic or diagnostic targets, particularly if they contribute to fibril nucleation or structural stability.

Our findings demonstrate that proteins identified in ischemic stroke thrombi consistently score highly for predicted amyloidogenicity, regardless of stroke subtype. This supports the experimental observation that amyloid-forming potential may contribute to thrombus structure or stability *in vivo*. The application of sequence-based tools such as AmyloGram provides a complementary strategy for identifying these features, especially in cases where traditional proteomic or histological methods may not detect underlying amyloidogenic signatures. These results motivate further investigation into the structural roles of amyloid-prone proteins in thrombotic disease and may inform future therapeutic or diagnostic approaches.

## Materials and methods

### Amyloidogenicity prediction using AmyloGram

AmyloGram [53,54] was used to estimate the amyloid-forming potential of protein sequences. This tool employs overlapping n-gram encoding of amino acid sequences and random forest classification to assign a global score ranging from 0 to 1, reflecting the likelihood of amyloidogenicity. It also produces residue-level profiles showing localized amyloidogenic potential across the protein sequence (some examples in [24] and below).

### Reference dataset of verified amyloid proteins

To calibrate the AmyloGram scoring framework, we analyzed 204 proteins tagged as amyloidogenic in UniProt and marked as manually reviewed. (The specific query is https://www.uniprot.org/uniprotkb?query=%28keyword%3AKW-0034%29&facets=reviewed%3Atrue) This dataset included entries from 95 species, with 24 annotated as human proteins. For all threshold-based analyses and clinical interpretation, we restricted comparative calibration and scoring to the 24 human proteins, given their greater biological and translational relevance. We recognize that some known amyloidogenic human proteins do not appear, but as our chief purpose was to establish the kinds of values in Amylogram that light up known amyloids, our purposes are adequately served. Canonical full-length FASTA sequences were retrieved. Isoforms, fragments, and signal peptide trimming were excluded. Proteins were submitted in batches of approximately 40 to the AmyloGram web server http://biongram.biotech.uni.wroc.pl/AmyloGram/, and global scores were recorded and compiled for downstream comparison (Supplementary Table S1).

### Additional analysis using the R version of AmyloGram

The UniProt version of the entire human proteome (39,675 kb) was downloaded from https://www.uniprot.org/uniprotkb?query=proteome:UP000005640 on 1/7/25 and consisted of 83,587 individual FASTA sequences. These were analyzed using the R version of AmyloGram https://github.com/michbur/AmyloGram. A very small subset of 20 proteins did not run (for reasons that were unclear), leaving a total of 83,567; since this represents 99.98% of them, we do not consider that the loss of 20 will have any effect on the conclusions drawn. Default parameters were used for all analyses.

### Stroke thrombus proteomic datasets

We applied the same scoring method to proteins identified in thrombi from patients with AIS. Two mass spectrometry-based datasets were used. The first included 14 proteins found to be enriched in thrombi from patients with cardioembolic stroke [88]. The second included six proteins reported to be more abundant in thrombi from patients with atherothrombotic stroke compared with those with cardioembolic stroke [89]. Protein names or gene symbols were mapped to their corresponding UniProt accessions, and canonical human sequences were retrieved for analysis.

### Score threshold and comparative analysis

AmyloGram scores were interpreted relative to the calibration set. A threshold of 0.7 was used to indicate high amyloid-forming potential, based on its consistent alignment with verified amyloidogenic proteins in the UniProt set. Proteins from stroke thrombi were evaluated individually, and their scores were compared across the two stroke subtypes to assess whether thrombus type was associated with predicted amyloidogenicity.

### Software and data management

Protein identifiers, sequence retrieval, and result collation were managed using Microsoft Excel. UniProt IDs, AmyloGram scores, and relevant metadata are included in Supplementary Tables 1; 2.

## Supplementary material

Online supplementary table 1

Online supplementary table 2

## Data Availability

All data supporting the findings of this study are available within the article and its supplementary materials. The UniProt human protein dataset used for calibration is publicly accessible at https://www.uniprot.org/uniprotkb?query=(keyword:KW-0034)&facets=reviewed:true. Stroke thrombus proteomic datasets were obtained from previously published studies [88,89] and are cited accordingly. Amyloidogenicity scores for all analyzed proteins, as generated by the online AmyloGram server (http://biongram.biotech.uni.wroc.pl/AmyloGram/), are provided in Supplementary Table 1, and those from the R program in Supplementary Table 2.

## Open Access

This article has been published open access under our Subscribe to Open programme, made possible through the support of our subscribing institutions, learn more here: https://portlandpress.com/pages/open_access_options_and_prices#conditional

## Abbreviations

AIS: acute ischemic stroke
LVO: large vessel occlusion
